# A Rare Case of Plasmablastic Lymphoma With Difficult Diagnosis and Treatment in a Cardiac Transplant Patient

**DOI:** 10.7759/cureus.98480

**Published:** 2025-12-04

**Authors:** Owais Gul, Muhammad Abdul Basit, Saqib Gul, Obuli Srinivasan Gurunathan, Maria Khalid

**Affiliations:** 1 Internal Medicine, United Health Services Wilson Medical Center, Johnson City, USA; 2 Internal Medicine, Hamdard College of Medicine and Dentistry, Karachi, PAK

**Keywords:** bone marrow, bortezomib, daratumumab, epoch, epstein-barr virus, hiv, plasmablastic lymphoma (pbl), plasmacytic myeloma, post-transplant lymphoproliferative disorder

## Abstract

Plasmablastic lymphoma (PBL) is a rare lymphoproliferative disease that can affect immunocompromised post-transplant patients. Patients with positive human immunodeficiency virus (HIV) and Epstein-Barr virus (EBV) status are at high risk. PBL shares many immunological markers and cytological characteristics with plasmacytic myeloma, which can lead to uncertainty and delay in diagnosis. However, myeloma typically involves the bone marrow, and the presence of other laboratory findings in myeloma, such as paraproteinemia, renal disease, and hypercalcemia, along with careful review of the biopsy, may help to distinguish between the two conditions. Treatment options are limited with poor prognosis and survival; however, intensive chemotherapy regimens such as EPOCH (etoposide, prednisone, vincristine, cyclophosphamide, and doxorubicin) are often used, sometimes in combination with agents that are being traditionally used in the treatment of myeloma, such as bortezomib and daratumumab. Here, we describe the case of a 54-year-old male patient who developed post-transplant lymphoproliferative disorder (PTLD) after undergoing cardiac transplantation with biopsy findings suspicious for both PBL and plasmacytic myeloma and treated with rituximab plus the EPOCH regimen, bortezomib, and daratumumab.

## Introduction

Plasmablastic lymphoma (PBL) is a rare and aggressive B-cell lymphoma that primarily affects immunocompromised individuals, including those with human immunodeficiency virus (HIV) or solid organ transplants. It is characterized by the proliferation of immature plasma cells and has a poor prognosis. The neoplastic cells exhibit a high proliferation index, characterized by the presence of plasma cell markers and the absence or minimal expression of B-cell markers [1]. Distinguishing between the post-transplant lymphoproliferative disorders (PTLDs) can be challenging due to the overlapping clinical presentations and immunological profiles. One such entity is plasmacytic myeloma, which shares many immunological markers and cytological characteristics with PBL. However, involvement of the bone marrow in myeloma and positivity status for Epstein-Barr virus (EBV) or HIV in the majority of PBL cases may help in the differentiation between the two conditions [2]. This case is unique due to the manifestation of PBL in a cardiac transplant patient, which is of particularly rare occurrence given the relatively low number of such transplants compared to other solid organ transplants.

## Case presentation

**Figure 1:**
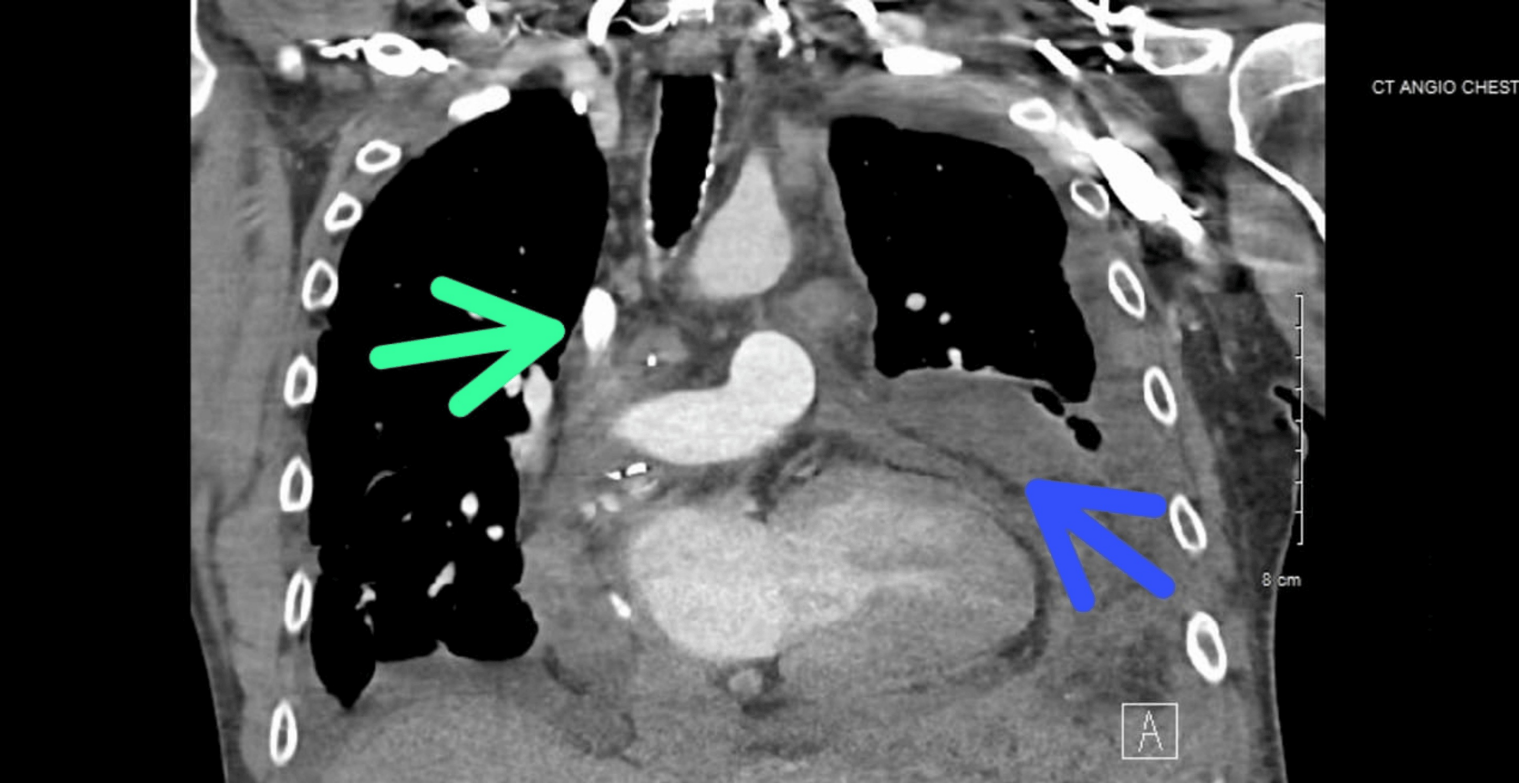
Coronal view of CT angiogram of the chest showing mediastinal lymphadenopathy (green arrow) and left subpleural opacity (blue arrow) CT: computed tomography

**Figure 2:**
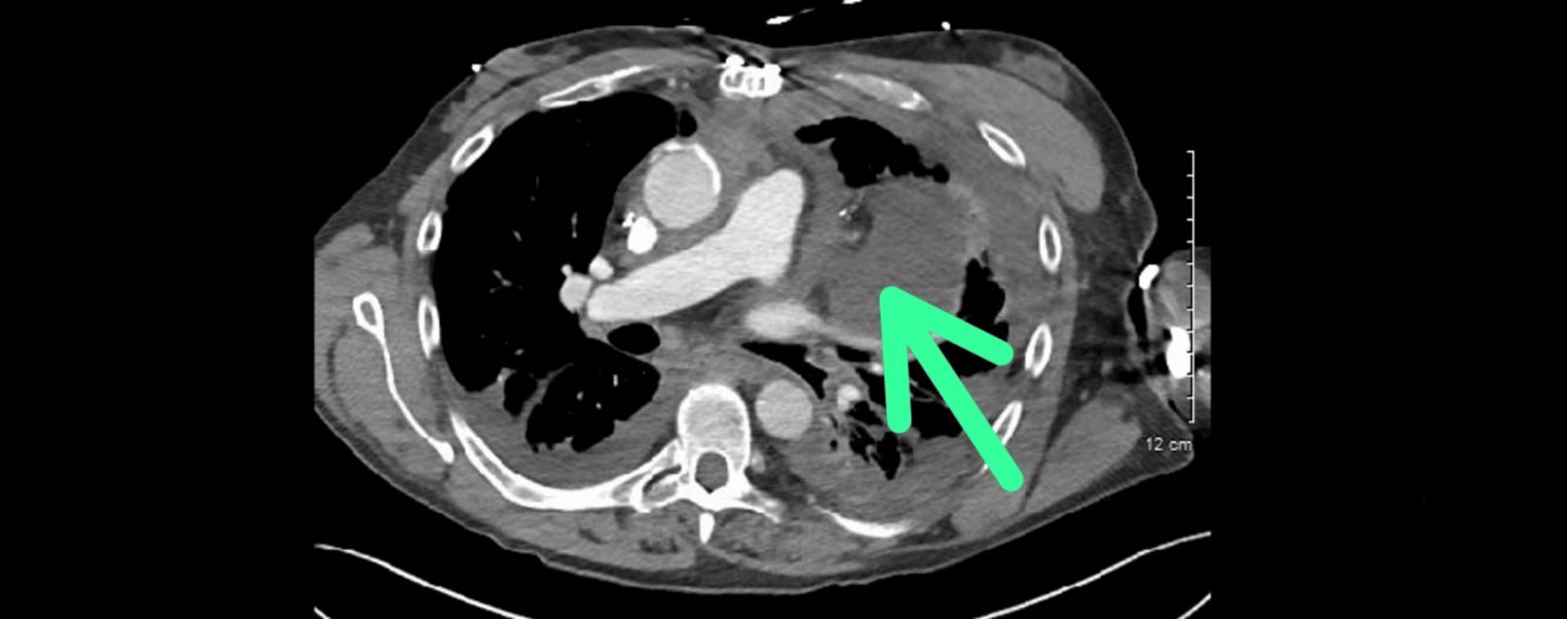
Cross-sectional view of CT angiogram of the chest showing opacities originating from the left pericardial fat (green arrow) CT: computed tomography

A 54-year-old man presented to the emergency department with complaints of shortness of breath on exertion for the past seven days. His past medical history was significant for iron deficiency anemia, dyslipidemia, and generalized anxiety disorder. He also underwent an orthotopic heart transplant in the past (about 10 months ago) due to a history of ischemic cardiomyopathy with severely reduced left ventricular ejection fraction complicated by sustained ventricular tachycardia. The patient, unfortunately, had to undergo the heart transplant twice at that time, as the first one failed, and he ended up being on extracorporeal membrane oxygenation (ECMO) for 4-5 days, which led to gangrenous necrosis of his bilateral upper and lower extremities, for which he underwent extensive amputations. The patient had been on chronic immunosuppressive therapy with tacrolimus for the past several months and was regularly following up with the heart transplant team in the outpatient setting. He denied any fevers, chills, cough, nausea, vomiting, paroxysmal nocturnal dyspnea (PND), orthopnea, or leg swelling. His laboratory workup, including complete blood count and comprehensive metabolic panel, was negative for any abnormalities. Serological studies revealed negative results for HIV-1 and HIV-2, hepatitis B surface antigen, and anti-hepatitis C. Serology was also obtained for EBV and cytomegalovirus (CMV). CMV quantitative DNA by polymerase chain reaction (PCR) showed undetectable copies, but EBV DNA came back positive at 37.2 IU/mL (normal <35 IU/mL). He underwent a computed tomography (CT) scan of the chest with angiogram that ruled out pulmonary embolism but showed multinodular opacities within the left pericardial fat and left subpleural space with extensive fat strandings within the involved regions. Mild mediastinal lymphadenopathy was also noticed, raising suspicion for PTLD (Figures 1-2).

The patient then underwent thoracotomy and biopsy of the largest mediastinal lymph node, which came back positive for diffuse large B-cell lymphoma (DLBCL). Flow cytometry revealed a small lambda-restricted B-cell population expressing various cluster of differentiation (CD) markers, including CD22, CD19, CD45, CD138, and CD38, but negative for CD5, CD10, CD20, CD23, CD56, and terminal deoxynucleotidyl transferase (TdT) markers. A high Ki-67 mitotic index of more than 80% was noticed, suggestive of marked cellular proliferation. Following this, a bone marrow biopsy was performed, which came back negative for any abnormal cell population, hence ruling out bone marrow involvement with the disease. Serum protein electrophoresis (SPEP) was done, which showed no monoclonal protein spike (M-spike).

The patient was started on chemotherapy with the R-EPOCH (rituximab, etoposide, prednisone, vincristine or oncovin, cyclophosphamide, and doxorubicin) regimen for DLBCL, a prototype of PTLD, based on the mediastinal lymph node biopsy results. Chemotherapy had to be interrupted temporarily due to the development of tumor lysis syndrome, but was restarted later. Further review of the pathology revealed that the initial mediastinal lymph node biopsy appeared to be suspicious for plasmacytic myeloma, so the chemotherapeutic regimen was changed to the CyBorD (cyclophosphamide, bortezomib, and dexamethasone) regimen. Daratumumab was also added, and the patient underwent a lumbar puncture with intrathecal methotrexate therapy for the prophylaxis of central nervous system (CNS) metastasis of the disease. Cerebrospinal fluid (CSF) flow cytometry was negative for any disease. The bone marrow biopsy was repeated and again showed no bone marrow involvement.

**Figure 3:**
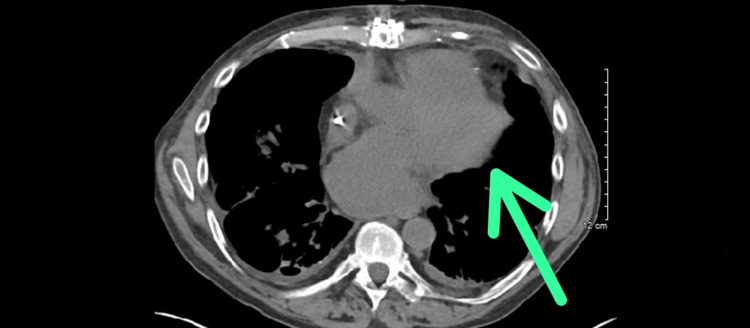
Cross-sectional view of the repeat CT of the chest without contrast showing improvement in the previously seen left pericardial fat opacity (green arrow) CT: computed tomography

The patient's symptoms responded well to the changed regimen and with the addition of daratumumab after finishing two cycles. His repeat CT scan of the chest without contrast showed marked improvement in the left pericardial fat opacities that were identified before (Figure 3).

The patient was recommended to continue and complete the third cycle of the combined regimen, which was delayed due to the development of leukopenia, thrombocytopenia, and *Clostridium difficile* infection. The rest of the laboratory workup was negative for the presence of any hypercalcemia or kidney disease. He was then referred to another tertiary care oncology center for a second opinion regarding his chemotherapeutic regimen and review of his initial biopsy findings.

## Discussion

PBL can be challenging to differentiate from plasmacytic myeloma due to the shared origin of cells. Distinguishing factors include that myeloma typically involves the bone marrow, is characterized by renal disease, paraproteinemia, osteolytic lesions, and hypercalcemia, and lacks associations with HIV and EBV [1]. Our patient's presentation, with numerous nodular opacities on the chest CT, pleural effusions, mediastinal and subpleural masses, and no marrow involvement, supported a PBL diagnosis. Additionally, the patient was receiving immunosuppressive treatment and had recently received a heart transplant, two major risk factors for PBL and other PTLDs. 

The tumor cells in PBL express plasma cell markers, such as CD38 and CD138, and frequently lack the traditional B-cell markers, namely, CD20 and PAX5. This immunological profile overlaps with that of myeloma, especially in cases showing plasmablastic differentiation on cytology. The morphology of the cells is another crucial diagnostic factor. Plasmablasts, which are young blast-like plasma cells with significant mitotic activity and an elevated Ki-67 proliferation index greater than 80%, are the hallmark of PBL. In contrast, plasmacytic myeloma cells are typically less proliferative and more mature [2]. In this case, the pathology report revealed plasmablastic morphology and a high Ki-67 index, which further supported the diagnosis of PBL over myeloma. 

Treatment options for PBL are not well-established. Chemotherapeutic regimens are commonly used, sometimes combined with antiretroviral therapy in case of associated HIV infection. The National Comprehensive Cancer Network (NCCN) recommends the use of the CHOP (cyclophosphamide, doxorubicin, vincristine, and prednisone) regimen or CHOP-like regimens including Hyper-CVAD-MA (hyperfractionated cyclophosphamide, vincristine, doxorubicin, dexamethasone, and high-dose methotrexate and cytarabine), CODOX-M/IVAC (cyclophosphamide, vincristine, doxorubicin, high-dose methotrexate/ifosfamide, etoposide, and high-dose cytarabine), and EPOCH (etoposide, prednisone, vincristine, cyclophosphamide, and doxorubicin) [3]. Treatment with EPOCH led to more favorable overall survival (OS) than CHOP alone in univariate and multivariate analysis; therefore, NCCN favors intensive regimens like EPOCH over CHOP for PBL. However, the effectiveness of rituximab becomes highly questionable in the case of a lack of CD20 expression by the tumor cells [4]. Bortezomib has been classically used in the treatment of multiple myeloma, but, because of the shared cellular origin between myeloma and PBL, it has become one of the most frequently used agents in the treatment of PBL. Bortezomib can be used alone or in combination with chemotherapy, such as with EPOCH (V-EPOCH), cyclophosphamide, dexamethasone, or lenalidomide. Several case reports and series have reported improved outcomes in both HIV-positive and HIV-negative PBL-affected patients with bortezomib when combined with the rest of the chemotherapeutic regimens [5]. Our patient initially received R-EPOCH for DLBCL, but the treatment was changed to Dara-CyBorD after the review of pathology was suspicious for plasmablastic myeloma. 

However, survival rates for PBL remain low, with a five-year survival rate of less than 30%, and the median OS was noted to be between nine and 15 months. This is in contrast to multiple myeloma, with a five-year survival rate of more than 60%. This has led to the use of novel agents such as daratumumab, which were being used mainly in the treatment of multiple myeloma, due to similarities noted between PBL cells and myeloma cells. Daratumumab is a monoclonal antibody directed against CD38, and it has shown promising results in recent studies in terms of controlling the disease progression when used with EPOCH or other chemotherapeutic regimens [6].

The guidelines from NCCN also recommend the use of intrathecal methotrexate for CNS prophylaxis in all HIV patients who develop lymphoma. Patients with PBL can be treated for CNS prophylaxis due to a strong link to HIV disease, high risk of proliferation, and more extranodal involvement. Our patient was HIV negative but showed evidence of extranodal disease with high proliferation risk, so he was treated with intrathecal methotrexate. Autologous stem cell transplant (ASCT) can also be considered either as an option in the first-line treatment in addition to chemotherapy or during relapse. However, data is limited to case reports only with regard to allogeneic stem cell transplants [4].

This case highlights the importance of timely diagnosis of PTLD as some of them, such as PBL, carry a poor prognosis. Additionally, it emphasizes the importance of morphologic analysis, immunophenotyping, EBV testing, and clinical features in differentiating PBL from other lymphoproliferative diseases. Multidisciplinary coordination involving hematopathologists, oncologists, and transplant specialists is crucial due to the overlap in the diagnostic findings. 

## Conclusions

PBL is an aggressive B-cell lymphoma that affects post-transplant patients. Plasmacytic myeloma can mimic PBL because of the sharing of immunological markers on flow cytometry. Factors such as EBV positivity and a high Ki-67 proliferative index exhibited by plasmablastic cells, along with sparing of the bone marrow, supported the diagnosis of PBL in our case. Laboratory manifestations, including renal disease, paraproteinemia, and hypercalcemia, are more common in myeloma, and these were not present in our patient. In most cases, careful review of the biopsy and a multidisciplinary approach are required to distinguish between the two conditions, which is important as the survival rate of PBL is significantly lower compared to myeloma and treatment options are limited. Intensive chemotherapy regimens are commonly deployed, but recently the use of myeloma-specific drugs such as bortezomib and daratumumab has also increased due to similarities noticed between myeloma cells and plasmablasts.
